# Highly Thermal Stable Phenolic Resin Based on Double-Decker-Shaped POSS Nanocomposites for Supercapacitors

**DOI:** 10.3390/polym12092151

**Published:** 2020-09-21

**Authors:** Wei-Cheng Chen, Yuan-Tzu Liu, Shiao-Wei Kuo

**Affiliations:** 1 Department of Materials and Optoelectronic Science, Center of Crystal Research, National Sun Yat-Sen University, Kaohsiung 80424, Taiwan; chwei566@gmail.com (W.-C.C.); ruby860420@gmail.com (Y.-T.L.); 2 Department of Medicinal and Applied Chemistry, Kaohsiung Medical University, Kaohsiung 807, Taiwan

**Keywords:** POSS, phenolic resin, hydrogen bonding, supercapacitors

## Abstract

In this study we incorporated various amounts of a double-decker silsesquioxane (DDSQ) into phenolic/DDSQ hybrids, which we prepared from a bifunctionalized phenolic DDSQ derivative (DDSQ-4OH), phenol, and CH_2_O under basic conditions (with DDSQ-4OH itself prepared through hydrosilylation of nadic anhydride with DDSQ and subsequent reaction with 4-aminophenol). We characterized these phenolic/DDSQ hybrids using Fourier transform infrared spectroscopy; ^1^H, ^13^C, and ^29^Si nuclear magnetic resonance spectroscopy; X-ray photoelectron spectroscopy (XPS); and thermogravimetric analysis. The thermal decomposition temperature and char yield both increased significantly upon increasing the DDSQ content, with the DDSQ units providing an inorganic protection layer on the phenolic surface, as confirmed through XPS analyses. We obtained carbon/DDSQ hybrids from the phenolic/DDSQ hybrids after thermal curing and calcination at 900 °C; these carbon/DDSQ hybrids displayed electrochemical properties superior to those of previously reported counterparts.

## 1. Introduction

Compared with inorganic materials, organic polymers usually possess lower moduli and poorer thermal stabilities and mechanical properties. The incorporation of inorganic nanoparticles (NPs) nanotubes, or nanosheets (e.g., clay [1,2]), graphene [3,4,5], carbon nanotubes [6,7,8], or polyhedral oligomeric silsesquioxanes (POSS) [9,10,11,12,13] into a polymer matrix is, however, a relatively simple approach toward improving its mechanical and thermal properties.

Phenolic resin is a typical thermosetting resin that has many applications (e.g., in laboratory countertops, adhesives, and coatings) because of its structural integrity and solvent-resistance. Nevertheless, phenolic resin has been neglected as a material for use in polymer nanocomposites because it has a three-dimensional structure even when it is not crosslinked. Several approaches have been developed, however, to overcome this problem to enhance thermal stability [14,15,16,17,18,19,20,21,22,23,24,25]. For example, phenolic nanocomposites have been synthesized through intercalative polymerization in organic-modified clays [14] and through sol–gel reactions [15,16]. In addition, the ability of POSS to improve the thermal properties of phenolic resin have been discussed widely [17,18,19,20,21,22,23,24,25]. Indeed, we have prepared several phenolic/POSS nanocomposites incorporating various functionalized (e.g., isobutyl, acrylate, and acetoxystyrene) POSS NPs, stabilized through hydrogen bonding [17,18,19,20,21]. Zhang et al. incorporated multi-functionalized amine POSS NPs into a phenolic resin, stabilized through hydrogen bonding and covalent crosslinking, to enhance its mechanical properties [22]. Zheng and Liu et al. used a multi-functionalized epoxy POSS as a crosslinking agent within phenolic resin, thereby improving its thermal stability [23,24]. Furthermore, we synthesized a multi-functionalized phenol POSS derivative and reacted it with phenol and CH_2_O to obtain various phenolic/POSS hybrids, which also exhibited high thermal stability and low surface energies [25].

Unfortunately, these phenolic/POSS nanocomposites prepared using multi-functionalized POSS NPs have led to insoluble cross-linked phenolic resins [22,23,24,25], restricting their applicability. When the POSS NPs presented more than two functional groups, the polymer/POSS nanocomposites formed crosslinked structures, even in the presence of only a small amount of the POSS NPs. Therefore, bifunctionalized POSS NPs, including double-decker silsesquioxane (DDSQ), that can form main-chain-type POSS units have allowed new approaches for the synthesis of novel polymer/POSS hybrids from, for example, polyurethane [26,27], polybenzoxazine [28,29,30], block copolymer [31,32], and polyimide [33,34] systems.

**Scheme 1:**
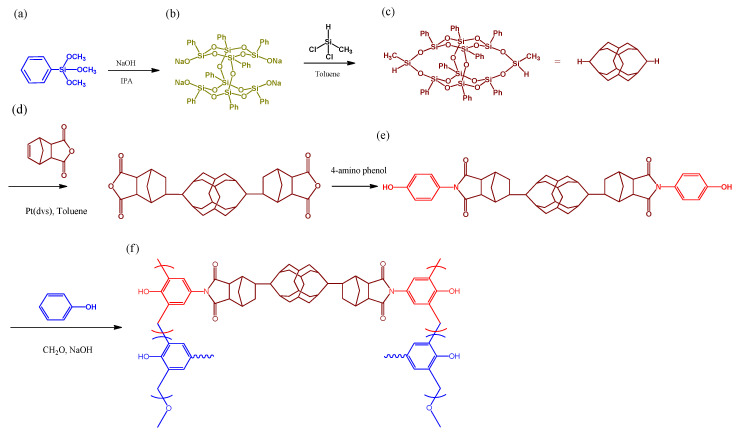
Chemical structures of (**a**) phenyltrimethoxysilane, (**b**) DD-Na, (**c**) double-decker silsesquioxane (DDSQ), (**d**) DDSQ-nadic anhydride (ND), and (**e**) DDSQ-4OH and (**f**) the preparation of phenolic/DDSQ (PDDSQ) hybrids from DDSQ-4OH and phenol under basic conditions.

In this study, we synthesized the bifunctionalized phenolic DDSQ derivative DDSQ-4OH (Scheme 1e) through the hydrosilylation of nadic anhydride (ND) with DDSQ (itself obtained from phenyltrimethylsilane (Scheme 1a) and DD-Na (Scheme 1b), giving DDSQ-ND (Scheme 1d), and subsequent reaction with 4-aminophenol. We then prepared phenolic/DDSQ (PDDSQ) hybrids incorporating various amounts of DDSQ through the reactions of DDSQ-4OH with phenol and CH_2_O under basic conditions (Scheme 1f). Furthermore, we obtained carbon/DDSQ hybrids after thermal curing and calcination of these PDDSQ hybrids; these carbon materials displayed electrochemical properties superior to those of related non-carbonized and even carbonized materials.

## 2. Materials and Methods

### 2.1. Materials

Phenyltrimethoxysilane (Scheme 1a), sodium hydroxide (NaOH), methyl dichlorosilane (CH_3_Cl_2_SiH), charcoal, acetonitrile, ND, paraformaldehyde (CH_2_O), platinum divinyltetramethyldisiloxane complex (Pt (dvs)), 4-aminophenol, and phenol were purchased from Sigma–Aldrich (St. Louis, MO, USA). DDSQ-4OH (Scheme 1e) was synthesized according to a method described previously [18,35].

### 2.2. PDDSQ hybrids

Mixtures of phenol and various amounts of DDSQ-4OH in dioxane and aqueous NaOH solution were stirred for 10 min; formaldehyde in water was added and then the mixtures were stirred for 10 min before heating at 70 °C for 1 h. All compositions of these PDDSQ hybrids used in this study were summarized in Table 1. The mixture was cooled to room temperature and treated with dilute HCl solution to pH 7.0. The water content in the viscous liquid was decreased through distillation under reduced pressure for 2 h at 45 °C. The resulting PDDSQ hybrids were dissolved in tetrahydrofuran (THF) and stirred vigorously; the precipitated NaCl was removed through centrifugation.

### 2.3. Carbon/DDSQ Hybrids

A PDDSQ hybrid was stirred in THF until it had dissolved completely. The solution was poured into a Teflon pan and then the solvent was evaporated at room temperature over 2 days. The resulting solid was heated at 150 °C for 24 h to ensure complete thermal curing. Finally, the PDDSQ hybrid was subjected to thermal treatment at 900 °C for 6 h under a N_2_ atmosphere in a tubular furnace to give a carbon/DDSQ hybrid.

## 3. Results and Discussion

### 3.1. Synthesis of the Pure PDDSQ Hybrid

**Figure 1:**
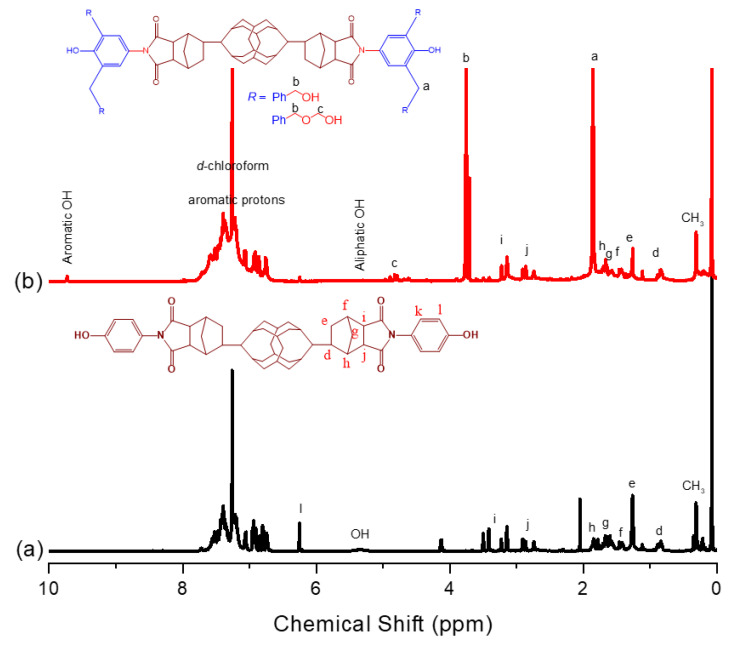
^1^H NMR spectra of (**a**) DDSQ-4OH and (**b**) pure PDDSQ.

Scheme 1 displays our approach for the synthesis of PDDSQ hybrids featuring various contents of DDSQ; we confirmed their structures using gel permeation chromatography apparatus (GPC, Waters 510, Labx, Midland, ON, Canada) and Fourier transform infrared (FTIR, Nicolet iS50, Thermo Fisher Scientific, Waltham MA, USA) and nuclear magnetic resonance (NMR, Bruker 500, Billerica, MA, USA) spectroscopy. First, we prepared the PDDSQ hybrid from DDSQ-4OH, synthesized as presented in Scheme 1e,f, in the absence of phenol. Figure 1 provides the ^1^H NMR spectra of DDSQ-4OH and its corresponding pure PDDSQ. The spectrum of DDSQ-4OH (Figure 1a) featured a signal for the SiCH_3_ unit at 0.36 ppm and signals located between 7.14 and 7.50 ppm representing the aromatic protons of the DDSQ unit. Furthermore, signals from the aliphatic protons of the ND units appeared between 3.25 and 0.83 ppm, a broad signal appeared at 5.33 ppm for the OH units, and signals for the aromatic protons derived from 4-aminophenol appeared at 6.24 (*l*) and 6.80 (*k*) ppm, confirming the formation of DDSQ-4OH. The ^1^H NMR spectrum of the pure PDDSQ (Figure 1b) featured small signals at 5.30 and 9.73 ppm, representing its aliphatic and phenolic OH units, respectively. The spectrum of the pure PDDSQ also contained signals from various kinds of CH_2_ units: ArC**H**_2_Ar at 1.83 ppm (peak a), methylene ether (PhC**H**_2_OR) at 3.73 ppm (peak b), and methylol (OC**H**_2_OH) (peak c) at 4.82 ppm. Furthermore, the intensity of the signal of the aromatic protons from the 4-aminophenol unit at 6.24 ppm (*l*) had decreased significantly (Figure S1b), consistent with the formation of those bridging aromatic rings, ether, and methylol units.

**Figure 2:**
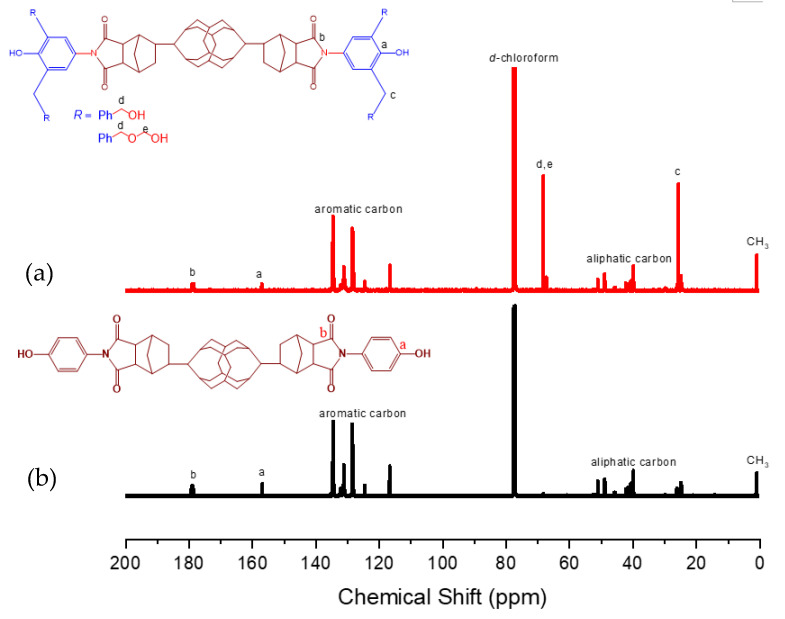
^13^C NMR spectra of (**a**) DDSQ-4OH and (**b**) pure PDDSQ.

Figure 2 displays the ^13^C NMR spectra of DDSQ-4OH and its corresponding pure PDDSQ. The spectrum of DDSQ-4OH (Figure 2a) featured a signal for the Si**C**H_3_ unit at 0.81 ppm, with signals located between 116.3 and 133.0 ppm representing aromatic carbon nuclei. The signals of the aliphatic carbon nuclei of the ND units appeared between 24.5 and 51.3 ppm and those of the aromatic C–OH (peak *a*) and C=O (peak *b*) units appeared at 157.3 and 178.8 ppm, confirming the formation of DDSQ-4OH. In the spectrum of the pure PDDSQ (Figure 2b), additional peaks appeared at 25.5 ppm for the Ar**C**H_2_Ar (peak c) units and at 68.1 ppm for the methylene ether (peak d) and methylol (peak e) units, along with the other signals from the DDSQ-4OH moieties, confirming the synthesis of the pure PDDSQ.

**Figure 3:**
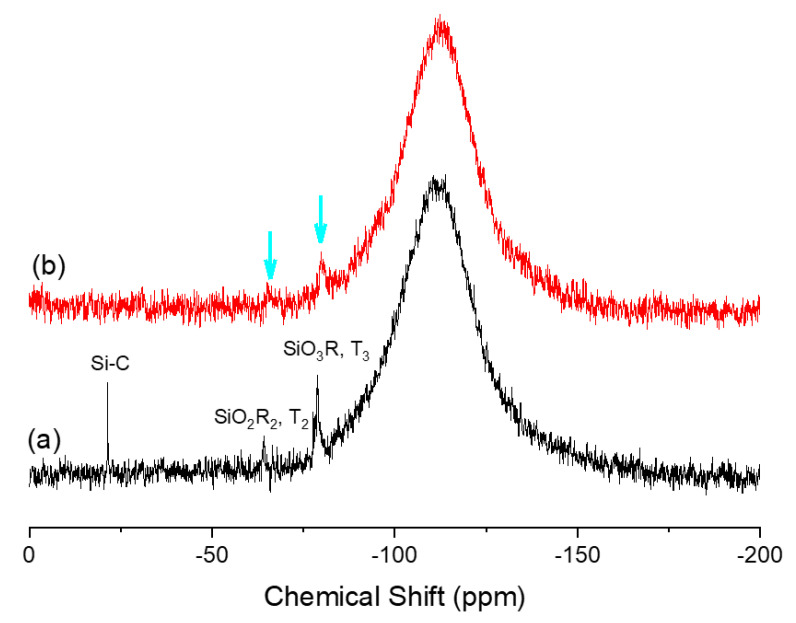
^29^Si NMR spectra of (**a**) DDSQ-4OH and (**b**) pure PDDSQ.

Figure S1 presents the FTIR spectra of DDSQ-4OH and its corresponding pure PDDSQ. The spectrum of DDSQ-4OH featured two strong signals at 1090 and 1130 cm^−1^, corresponding to the Si–O–Si and C–O units, as well as a signal at 1265 cm^−1^ representing the Si–CH_3_ groups. The spectrum of the pure PDDSQ did not feature any obvious additional peaks when compared with that of DDSQ-4OH, with only the intensity of the signal representing the OH groups having increased in intensity after the reaction of CH_2_O to form the pure PDDSQ. Figure 3 displays the ^29^Si NMR spectra of DDSQ-4OH and the pure PDDSQ. The spectrum of DDSQ-4OH featured three major peaks at –79.05, –64.17, and –21.34 ppm representing the SiO_3_R (T_3_), SiO_2_R_2_ (T_2_), and Si-C units, respectively (Figure 3a). In the spectrum of the pure PDDSQ (Figure 3b), two peaks at −79.05 and −64.17 ppm were remained, suggesting that the cage structure had not been destroyed; nevertheless, the intensities of these three peaks had decreased, as expected because the concentration of DDSQ units had decreased after the formation of the pure PDDSQ. The molecular weight of the pure PDDSQ (ca. 4500 g mol^−1^) was determined through GPC analysis, with a polydispersity index of 1.10 (Figure S2).

### 3.2. Synthesis of PDDSQ Hybrids

**Figure 4:**
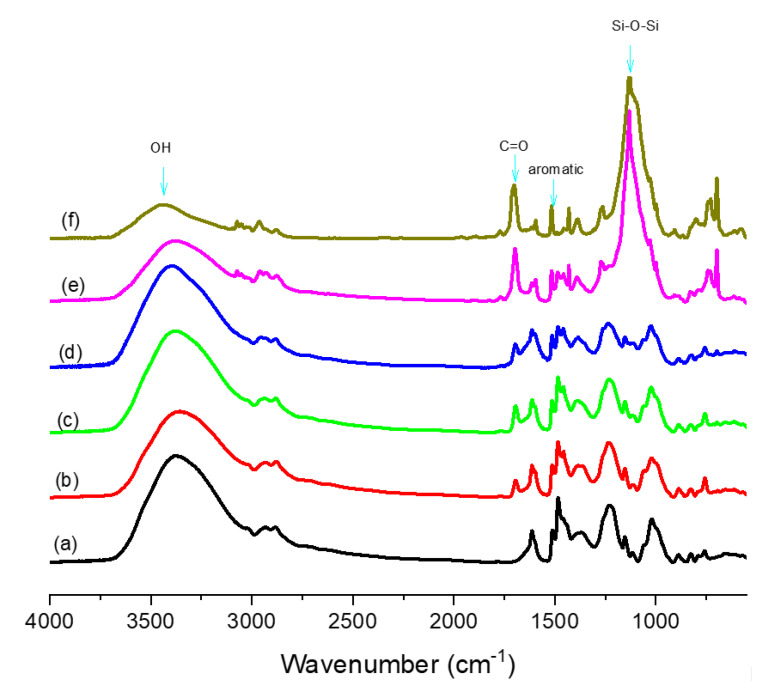
FTIR spectra of (**a**) pure phenolic, (**b**) PDDSQ-30, (**c**) PDDSQ-45, (**d**) PDDSQ-50, (**e**) PDDSQ-80, and (**f**) pure PDDSQ.

Having successfully synthesized the pure PDDSQ, we prepared PDDSQ hybrids incorporating various amounts of DDSQ, as displayed in Scheme 1f. Figure 4 presents FTIR spectra of these PDDSQ hybrids, recorded at room temperature. As mentioned above, the spectrum of the pure PDDSQ featured two strong signals at 1090 and 1265 cm^−1^ representing the Si–O–Si and Si–CH_3_ units of the DDSQ cage structure; in addition, two other obvious peaks appeared at 1702 and 1510 cm^−1^, representing the C=O groups and aromatic units of the ND and phenolic moieties, respectively. Here, we maintained the signal for the aromatic unit of the phenolic unit at 1510 cm^−1^, with the intensity of the signal at 1702 cm^−1^ for the C=O groups increasing upon increasing the concentration of DDSQ units in the PDDSQ hybrids. Thus, the composition of DDSQ could be determined readily by the peak ratio *A*_1702_/*A*_1510_ (Table 1). Furthermore, the spectrum of the pure phenolic resin displayed two major signals at 3375 and 3525 cm^−1^, corresponding to self-associated and free OH units, respectively. The intensity of the signal for the free OH units at 3525 cm^−1^ decreased upon increasing the DDSQ content in the PDDSQ hybrids from 20 to 100 wt %. The signal for the self-association of the phenolic OH units shifted to higher frequency upon increasing the concentration of DDSQ units in the PDDSQ hybrids, with the peak shifting to 3435 cm^−1^ as a result of the self-associative OH**···**OH bonding transforming into inter-associative OH**···**siloxane bonding. The frequency difference (∆*ν*) between the signals for the free and hydrogen-bonded OH groups can give a rough estimation of the average hydrogen bonding strength [36]. The average strength of the phenolic OH groups interacting with the DDSQ siloxane moieties (∆*ν* = 95 cm^−1^) appeared to be weaker than that for the self-associative OH**···**OH bonding of the pure phenolic resin (∆*ν* = 150 cm^−1^). This result is consistent with our previous finding that the inter-association equilibrium constant of phenolic/POSS (*K*_A_ = 38.7) is smaller than the self-association equilibrium constant of pure phenolic (*K*_B_ = 52.3), based on the Painter–Coleman association model [17,18]. Furthermore, Figure S3 presents ^1^H spectra of pure phenolic resin, PDDSQ-50 and pure PDDSQ recorded at room temperature. The peak assignment is similar with Figure 1 and we could observe that the PDDSQ hybrids are also synthesized successfully based on NMR and FTIR spectra analyses. 

**Figure 5:**
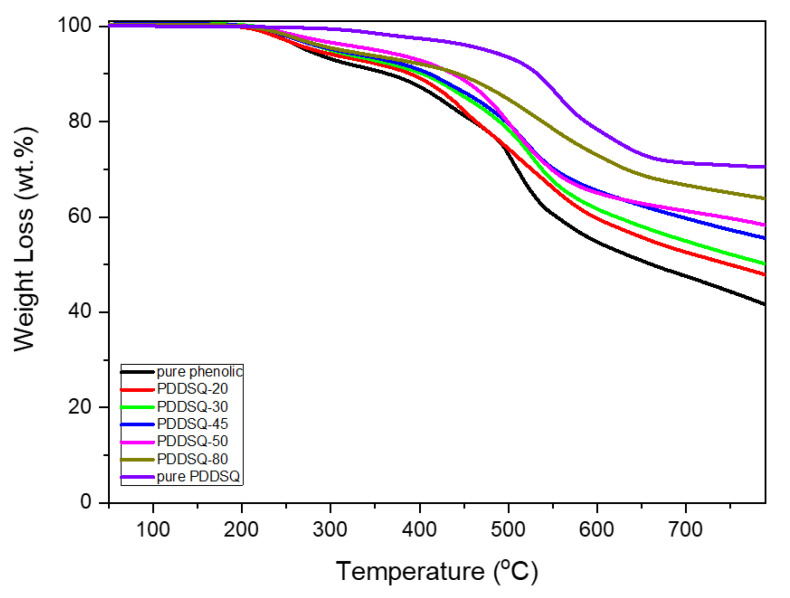
TGA analyses of PDDSQ hybrids containing various DDSQ contents.

**Figure 6:**
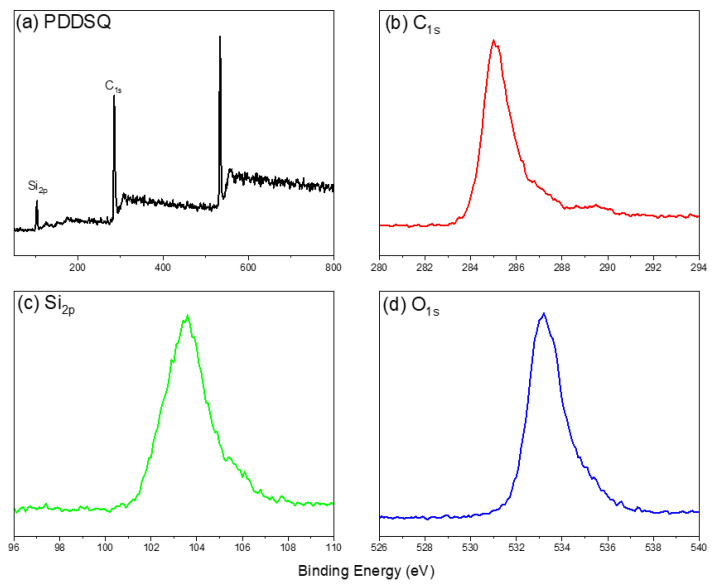
XPS analysis of (**a**) pure PDDSQ and its (**b**) C 1s, (**c**) Si 2p, and (**d**) O 1s atoms.

Although we had detected only weak intermolecular interactions between the phenolic OH groups and the DDSQ siloxane units, these interactions did still enhance the thermal stability of the PDDSQ hybrids, as determined using thermal gravimetric analyzer (TGA, Q-50, TA Instruments, New Castle, DE, USA). The TGA trace (Figure 5) of the pure phenolic resin revealed three thermal degradation steps, typical of the degradation of phenolic resin [37] and the derivative curve was displayed in Figure S4. The first step ca. 250 °C from the degradation of CH_2_ bridges, the second step at ca. 450 °C is due the broken of crosslinking network structure, and the third at ca. 500 ^o^C comes from the C–OH units in phenol were broken and then form H_2_ gas. Table 1 also summarizes the thermal degradation temperatures (*T*_d10_) and char yields at 800 °C of these PDDSQ hybrids. The char yields and values of *T*_d_ increased significantly upon increasing the DDSQ concentration, as expected. For example, the char yield and value of *T*_d_ increased by 12.2 wt% and 79 °C, respectively, for the PDDSQ-80 hybrid when compared with those of the pure phenolic resin. This behavior can be explained by considering the nano-reinforcement effect of incorporating inorganic DDSQ NPs into a phenolic matrix. The DDSQ units were presumably dispersed well within the phenolic resin at the nanoscale, stabilized through hydrogen bonding and covalent bonding, and, thus, they could enhance its initial decomposition temperature. Furthermore, the DDSQ units could retard the thermal motion of the polymer through the covalent bonding, and could also increase the chain spacing, giving rise to the lower thermal conductivity. It has been proposed that DDSQ units prefer to be oriented toward the air side in related nanostructures, because the low surface free energy of the siloxane units would screen out the polar functional units (e.g., carboxyl, OH, and urethane) [38,39]. As a result, we suspect that the DDSQ NPs in our PDDSQ hybrids would also have preferred to be oriented toward the air side, thereby forming an inorganic silica protection layer on the phenolic surface, resulting in higher thermal stability. To confirm this hypothesis, we used X-ray photoelectron spectroscopy (XPS) to examine the surface behavior of our PDDSQ hybrids (Figure 6). Figure 6a reveals the presence of three major elements: C atoms at 285.1 eV (Figure 6b), Si atoms at 103.6 eV (Figure 6c), and O atoms at 533.2 eV (Figure 6d). Large amounts of Si (20.0 wt %) and O (33.8 wt %) atoms were present at the surface, indicating that the DDSQ units had to migrated to the surface of the phenolic resin.

### 3.3. Synthesis of Carbon/DDSQ Hybrids for Electrochemical Applications

**Figure 7:**
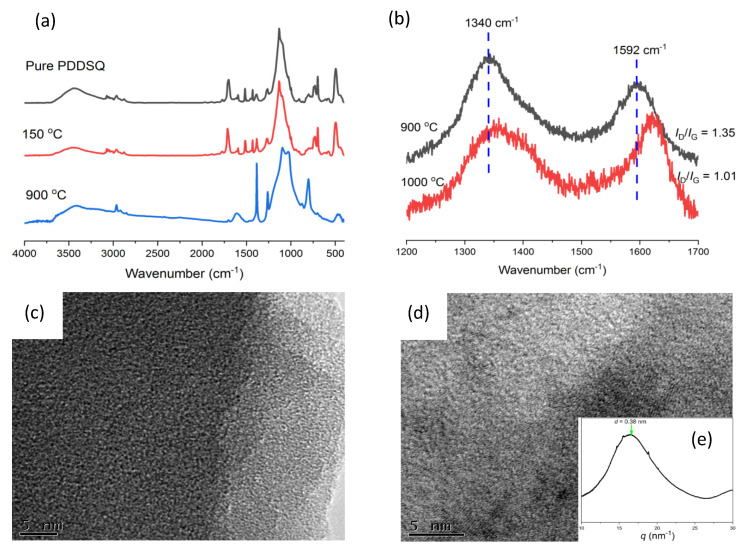
(**a**) FTIR spectra of pure PDDSQ before thermal treatment and after thermal curing at 150 and 900 °C. (**b**) Raman spectra of pure PDDSQ after thermal treatment at 900 and 1000 °C. (**c**,**d**) TEM images of pure PDDSQ after thermal treatment at (**c**) 900 and (**d**) 1000 °C. (**e**) corresponding WAXD pattern after thermal treatment at 1000 °C.

To obtain carbon/DDSQ hybrids with potential electrochemical applications, we heated the PDDSQ hybrids at 150 °C for 24 h to complete their thermal curing, and then subjected them to further heating at 900 or 1000 °C for 6 h under a N_2_ atmosphere. Figure 7a displays FTIR spectra of the pure PDDSQ before thermal curing, after thermal curing at 150 °C, and after heating at 900 °C to give its carbon/DDSQ hybrid. We have already discussed the spectrum of the pure PDDSQ (Figure S1); the spectrum of the sample obtained after thermal curing at 150 °C did not exhibit any major changes in its absorption peak. After thermal heating at 900 °C, however, the signals at 1701 cm^−1^ for the C=O groups and at 1432 cm^−1^ for the aliphatic CH_2_ units from the ND moieties both disappeared, and new peaks appeared at 1385 and 1612 cm^−1^ representing the carbon structure. Most importantly, the signals at 1090 cm^−1^ for the Si–O–Si units and at 1265 cm^−1^ for the Si–CH_3_ units were still present after thermal heating at 900 °C, suggesting that the DDSQ units remained dispersed within the carbon matrix in the carbon/DDSQ hybrids. Raman spectroscopy (HORIBA Jobin–yvon, T6400, Edison, NJ, USA) revealed the local graphitic structure of this carbon/DDSQ hybrid (Figure 7b), with two major carbonized structures identified by G- and D-bands representing sp^2^- and sp^3^-hybridized carbon atoms at 1592 and 1340 cm^−1^, respectively [40,41]. The G- and D-bands correspond to first- and second-order Raman scattering, respectively; this G-band had undergone a major shift from the wavenumber of a typical G-band (ca. 1582 cm^−1^), suggesting the presence of oxidized graphene after thermal treatment at 900 °C (1592 cm^−1^) or 1000 °C (1620 cm^−1^). Furthermore, the intensity ratio *I*_D_/*I*_G_ can be used to roughly characterize the degree of graphitization. The *I*_D_/*I*_G_ ratio (1.35 after treatment at 900 °C) decreased significantly to 1.01 after thermal treatment at 1000 °C, indicating a highly defected carbon structure containing Si, O, and N atoms in the former system, as confirmed by the Transmission electron microscope (TEM, JEOL, JEM-2100, Akishima, Japan) images in Figure 7c,d. The carbon/DDSQ hybrid after thermal treatment at 900 °C exhibited an irregular structure (Figure 7c), but its interlayer spacing was approximately 0.38 nm (based on the (002) plane of the carbon structure) in Figure 7d, confirmed by its WAXD pattern [42]. After thermal treatment at 1000 °C, the high value of *q* of 16.52 nm^−1^ (*d* = 0.38 nm) was due to the (002) plane of the carbon structure (Figure 7e). Therefore, the carbon/DDSQ hybrids had highly defected structures after thermal treatment at 900 °C; we selected three carbon materials—those prepared from the pure phenolic resin, PDDSQ-50, and the pure PDDSQ—to test their suitability for use as electrode materials in supercapacitors.

**Figure 8:**
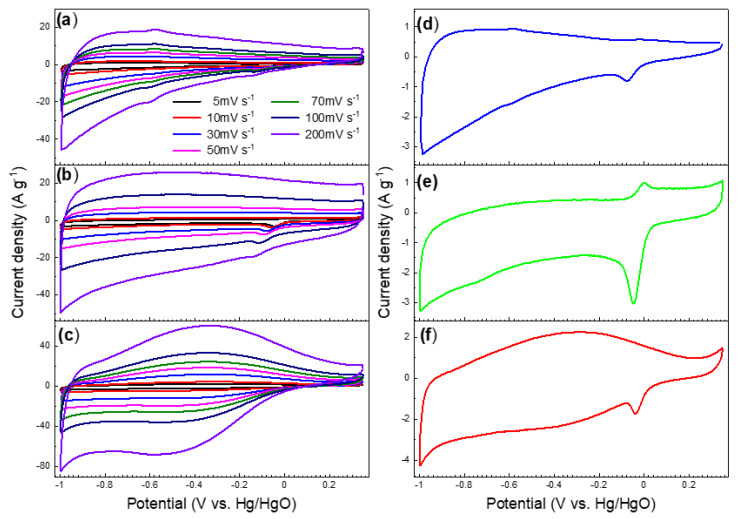
Cyclic voltammetry (CV) curves of carbon/DDSQ hybrids derived from (**a**,**d**) pure phenolic, (**b**,**e**) PDDSQ-50, and (**c**,**f**) pure PDDSQ, measured at (**a**–**c**) various scan rates and (**d**–**f**) a scan rate of 5 mV s^−1^ in 1 M KOH.

We used cyclic voltammetry (CV) and the galvanostatic charge/discharge (GCD) method in 1 M KOH as the aqueous electrolyte in a three-electrode system to investigate the electrochemical performance of our PDDSQ hybrids after thermal treatment at 900 °C. Figure 8a–c reveal quite different CV curves for the carbon/DDSQ hybrids derived from the pure phenolic, PDDSQ-50, and the pure PDDSQ, measured at sweep rates from 5 to 200 mV s^−1^ in a potential window from +0.35 to –1.00 V. The most distinct differences appeared at the lowest tested sweep rate of 5 mV s^−1^ (Figure 8d–f). The CV curve of the carbon material derived from pure phenolic (Figure 8d) had a rectangle-like shape, implying that its capacitive response resulted from electric double-layer capacitance (EDLC) [43,44]. The CV curve of the carbon material derived from PDDSQ (Figure 8f) had a quasi-rectangular shape featuring a hump, indicating that its capacitive response originated from pseudocapacitance, due to doping by heteroatoms (e.g., N and and O atoms), allowing a reversible redox process at the relatively low sweep rate (5 mV s^−1^) [45,46]. A similar phenomenon appeared in the CV curve of the carbon material derived from PDDSQ-50 (Figure 8e), suggesting a combination of pseudocapacitance and EDLC. Because of the many electroactive centers (e.g., N and O atoms and electron-rich phenyl rings) in the carbon material derived from the pure PDDSQ, reversible radical redox procedures occurred during the charging and discharging processes [47]. Furthermore, the current density of the carbon material derived from pure PDDSQ increased dramatically upon increasing the sweep rate, while the shape of its CV curve was retained (Figure 8c), indicating good rate capability and facile kinetics [48,49].

**Figure 9:**
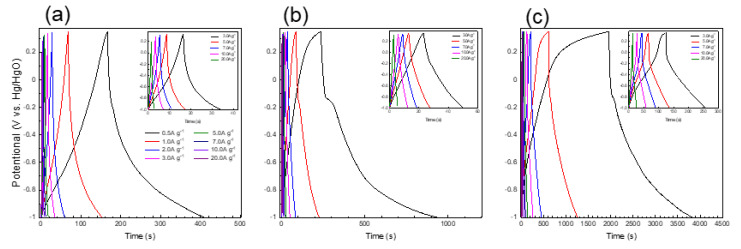
Specific capacitances of carbon/DDSQ hybrids derived from (**a**) pure phenolic, (**b**) PDDSQ-50, and (**c**) pure PDDSQ, measured at various current densities.

Figure 9 displays the GCD curves of the carbon/DDSQ hybrids derived from pure phenolic, PDDSQ-50, and pure PDDSQ, measured at current densities from 0.5 to 20 A g^−1^. The GCD curves of the carbon material derived from pure phenolic were triangular in shape, suggesting EDLC characteristics, while those of the carbon materials derived from PDDSQ-50 and pure PDDSQ were triangular with a slight bend, implying the presence of both pseudocapacitance and EDLC [49]. The discharging time of the carbon material derived from pure PDDSQ (Figure 9c) was much longer than that of the carbon material derived from pure phenolic (Figure 9a), suggesting that the former capacitance (pure PDDSQ) was larger than that of the latter. 

**Figure 10:**
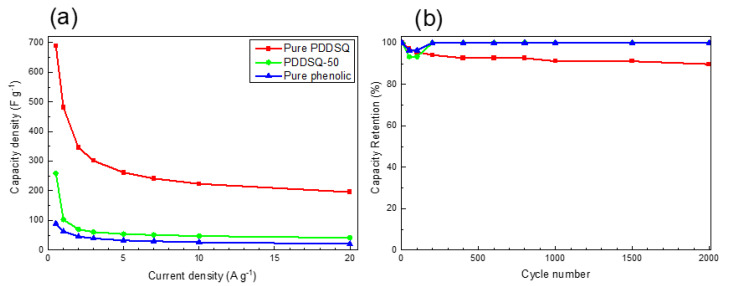
(**a**) specific capacitances measured at current densities from 0.5 to 10 A g^−1^ and (**b**) cycling performance measured at a current density of 10 A g^−1^ after 2000 cycles for the carbon/DDSQ hybrids derived from pure phenolic, PDDSQ-50, and pure PDDSQ.

We used Equation S1 to calculate the specific capacitances from the GCD curves (Figure 10). At a current density of 0.5 A g^−1^, the specific capacitance of the carbon material derived from pure PDDSQ (689.5 F g^−1^) was much larger than those of the carbon materials derived from PDDQ-50 (258.8 F g^−1^) and pure phenolic (89.9 F g^−1^). The excellent performance of the carbon material derived from pure PDDSQ was due to its many electroactive centers (e.g., N and O atoms and electron-rich phenyl rings) making it easier for the electrolyte to access the electrode surface [50]. In addition, the cage structure of DDSQ provided a large number of fine and interlaced channels for the free passage of electrolyte ions. After cycling over 2000 times at 10 A g^−1^, all of the carbon/DDSQ hybrids displayed high cycling stability, retaining over 90% of their original capacitances (Figure 10b). Although the carbon material derived from pure PDDSQ allowed the electrolyte ions to pass freely, a current density that is too large may cause many thermal effects, resulting in poorer durability. Interestingly, even though the specific capacitance of the carbon material derived from pure phenolic was relatively low, its stability was better than that of the carbon material derived from pure PDDSQ. After cycling the carbon material derived from PDDSQ-50 over 2000 times at 10 A g^−1^, it displayed close to 99.9% retention; thus, by adjusting the ratios of phenol and DDSQ-4OH during the copolymerization reaction we could balance the electrode materials to obtain both a large specific capacitance and high stability. 

**Figure 11:**
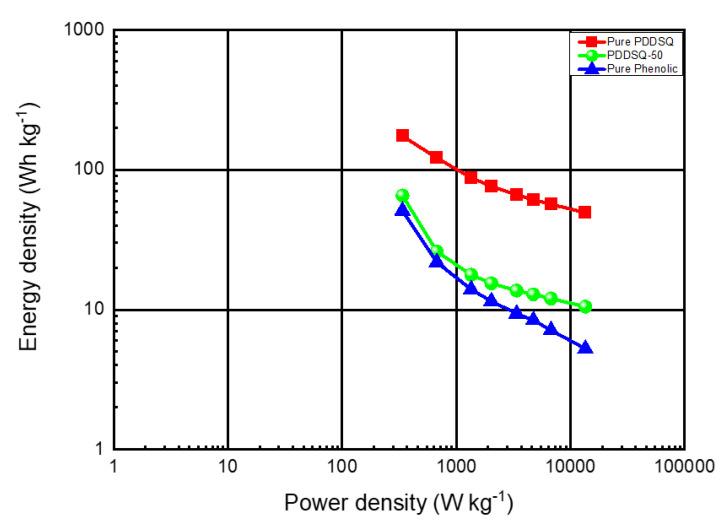
Ragone plots of the energy density and power density of the carbon/DDSQ hybrids derived from pure phenolic, PDDSQ-50, and pure PDDSQ.

Figure 11 displays the specific energy plotted with respect to the specific power. The carbon material derived from pure PDDSQ exhibited a maximum energy density of 174.5 W h kg^−1^ at a power density of 337.5 W kg^−1^; its energy density was maintained at 49.5 W h kg^−1^ even at a high power density of 13.5 kW kg^−1^. Compared with other materials prepared in previous studies, our present carbon/DDSQ hybrids displayed excellent properties and were much simpler to prepare, suggesting that they have potential for use as future electrode materials [50,51,52,53,54,55,56,57,58,59,60,61].

## 4. Conclusions

We have synthesized PDDSQ hybrids incorporating various amounts of DDSQ and confirmed their chemical structures using FTIR and NMR spectroscopy. All of these PDDSQ hybrids had char yields and thermal stabilities higher than those of the pure phenolic resin, and they increased upon increasing the DDSQ content in the hybrid systems. The DDSQ units provided an inorganic protection layer on the phenolic surface, as confirmed through XPS analysis. We obtained carbon/DDSQ hybrids after thermal curing and calcination of our PDDSQ hybrids; these carbon materials displayed electrochemical properties superior to those of previously reported counterparts.

## Figures and Tables

**Table 1:** Characterization data for the PDDSQ hybrids synthesized in this study.

Name	Monomer Feed (g)				DDSQ-4OH in hybrids (wt.%)	*T*_d_ (°C)	Char Yield (wt.%)	*M*_n_ (g/mol)
	Phenol	DDSQ-4OH	CH_2_O	NaOH	FTIR			
Pure Phenolic	10.0	-	17.25	4.25	0	364	41.6	5440
PDDSQ-20	9.0	1.0	15.72	3.87	20.2	389	47.7	4670
PDDSQ-30	4.0	1.0	7.09	1.75	32.3	403	49.7	4320
PDDSQ-45	2.3	1.0	4.22	1.04	44.9	411	55.5	4580
PDDSQ-50	1.5	1.0	2.78	0.68	50.7	438	58.2	4160
PDDSQ-80	1.0	1.0	1.92	0.47	80.5	443	63.8	4460
Pure PDDSQ	-	1.0	0.19	0.04	100	532	70.4	4500

## Supplementary Materials

The following are available online at https://www.mdpi.com/2073-4360/12/9/2151/s1, Table S1. Comparison between the energy density and power density data /specific capacitance of phenolic/DDSQ hybrids with different materials for supercapacitor application; Figure S1: FTIR spectra of (a) DDSQ-4OH and (b) pure PDDSQ; Figure S2: GPC analysis of pure PDDSQ; Figure S3: ^1^H NMR spectra of (a) pure phenolic, (b) PDDSQ-50 and (c) pure PDDSQ; Figure S4: TGA analyses of pure phenolic resin and its corresponding derivative curve.
