# Supplementary material for: Highly Thermal Stable Phenolic Resin Based on Double-Decker-Shaped POSS Nanocomposites for Supercapacitors
Source: Polymers (Basel). 2020 Sep 21;12(9):2151. doi: 10.3390/polym12092151 (PMC7570050; doi:10.3390/polym12092151)
Supplement: Supplementary file 1 [file polymers-12-02151-s001.pdf]

Supporting Information

Highly Thermal Stable Phenolic Resin Based on Double-Decker-Shaped POSS Nanocomposites for Supercapacitors

Wei-Cheng Chen[1], Yuan-Tzu Liu[1], and Shiao-Wei Kuo[1,2*]

[1]Department of Materials and Optoelectronic Science, Center of Crystal Research, National Sun Yat-Sen University, Kaohsiung, 80424, Taiwan. chwei566@gmail.com (W.C.C.) and m083100003@student.nsysu.edu.tw (Y.T.L.)

[2]Department of Medicinal and Applied Chemistry, Kaohsiung Medical University, Kaohsiung 807, Taiwan

Correspondence: kuosw@faculty.nsysu.edu.tw (S.-W.K.); Tel.: +886-7-525-4099 (S.-W.K.)

Characterization

FTIR spectroscopy was performed from 4000 to 400 cm^{-1} using a Nicolet iS50 FTIR spectrophotometer and the typical KBr pellet method. ^{1}H, ^{13}C and ^{29}Si NMR spectroscopy was performed using a Bruker 500 (500 MHz) spectrometer at 25 °C, with DMSO-d_6 and $CDCl_3$ as internal standards. The molecular weights of phenolic/DDSQ hybrids were evaluated from GPC (Waters 510 apparatus) where the molecular weight calibration was used by PS standard. TGA of the phenolic/DDSQ hybrid was performed using a TA Q-50 TGA analyzer; the sample was heated from 40 to 800 °C under a N_2 atmosphere at a heating rate of 20 °C min^{-1}. Raman spectra were recorded at room temperature using a Jobin-Yvon T6400 micro-Raman apparatus with a He–Cd laser as the excitation source (325 nm). WAXD data of all carbon/DDSQ samples were measured using the BL17A1 wiggler beamline of the National Synchrotron Radiation Research Center (NSRRC), Taiwan, with a wavelength (λ) of 1.33001 Å used from the monochromated beam based on a triangular bent Si (111) single crystal. TEM images of the carbon/DDSQ hybrids were recorded using a JEOL-JEM-2100 microscope operated at 200 kV.

Electrochemical Measurements

Working Electrode Cleaning: Prior to use, the glassy carbon electrode (GCE) was polished several times with 0.05-µm alumina powder, washed with EtOH after each polishing step, cleaned via sonication (5 min) in a water bath, washed with EtOH, and then dried in air.

Electrochemical Characterization: The electrochemical experiments were performed in a three-electrode cell using an Autolab potentiostat (PGSTAT204) and 1 M KOH as the aqueous electrolyte. The GCE was used as the working electrode (diameter: 5.61 mm; 0.2475 cm^2). A Pt wire was used as the counter electrode; Hg/HgO (RE-61AP, BAS) was used as the reference electrode. Slurries were prepared by dispersing the active material (45 wt. %), carbon black (45 wt. %), and Nafion (10 wt. %)

in EtOH (2 mL) and then sonicating for 1 h. A portion of this slurry (10 μL) was pipetted onto the tip of the electrode and dried in air for 30 min prior to use. The electrochemical performance was studied through CV at various sweep rates (from 5 to 200 mV s^{-1}) and through the GCD method in the potential range from 0.0 to –1.0 V (vs. Hg/HgO) at various current densities (from 2 to 20 A g^{-1}) in 1 M KOH as the aqueous electrolyte solution. The specific capacitances were calculated from the GCD data, using the following equation:

$$C_s = (I\Delta t)/(m\Delta V) \qquad \text{(S1)}$$

where C_s (F g^{-1}) is specific capacitance of the supercapacitor, I (A) is the discharge current, ΔV (V) is the potential window, Δt (s) is the discharge time, and m (g) is the mass of the carbon/DDSQ hybrids on the electrode. The energy density (E, Wh/kg), and the power density (P, W/kg) were calculated using the following equations:

$$E = 1000{*}C(\Delta V)^2/(2{*}3600) \qquad \text{(S2)}$$

$$P = E/(t/3600) \qquad \text{(S3)}$$

Table S1. Comparison between the energy density and power density data /specific capacitance of phenolic/DDSQ hybrids with different materials for supercapacitor application.

Materials	Specific Capacitance	Energy Density Wh/kg	Power Density kW/kg	Ref.
PDDSQ	689.5 F g^{-1} at 0.5A g^{-1}	121<br>49.5	0.68<br>13.60	This work
PDDSQ-50	258.9 F g^{-1} at 0.5A g^{-1}	26<br>10.5	0.68<br>13.60	This work
MnO_2	65 F g^{-1} at 0.25 A g^{-1}	8	28.00	51
RuO_2/graphene graphene	175 F g^{-1} at 0.5 A g^{-1}	19.7	6.8	52
MnO_2/CNTs CNTs	12.5 F g^{-1} at 0.14 A g^{-1}	42.0<br>28.0	0.48<br>19.3	53
Porous carbon derived from MOF	270 F g^{-1} at 2.0 A g^{-1}			54
Nitrogen-enriched Nanoporous Polytriazine	656 F g^{-1} at 1.0 A g^{-1}	102	1.6	50
Benzimidazole grafted graphene	410 F g^{-1} at 0.4 A g^{-1}	-	-	55
Nitrogen-doped graphitic carbon	255 F g^{-1} at 2 A g^{-1}	-	-	56
Pyrene-based covalent triazine frameworks	500 F g^{-1} at t 0.5 A g^{-1}	-	-	57
TPT-DAHQ	256 F g^{-1} at 0.5 A g^{-1}	43	1.36	58
TaPay-Py COF	209 F g^{-1} at 0.5 A g^{-1}	11.2	50.1	59
TFP-TPA COF	291.1 F g^{-1} at 2 A g^{-1}	49.5<br>28.4	1.36<br>13.6	60
NMCSs	416 F g^{-1} at 0.2 A g^{-1}	21.5<br>13.3	0.8<br>16	61

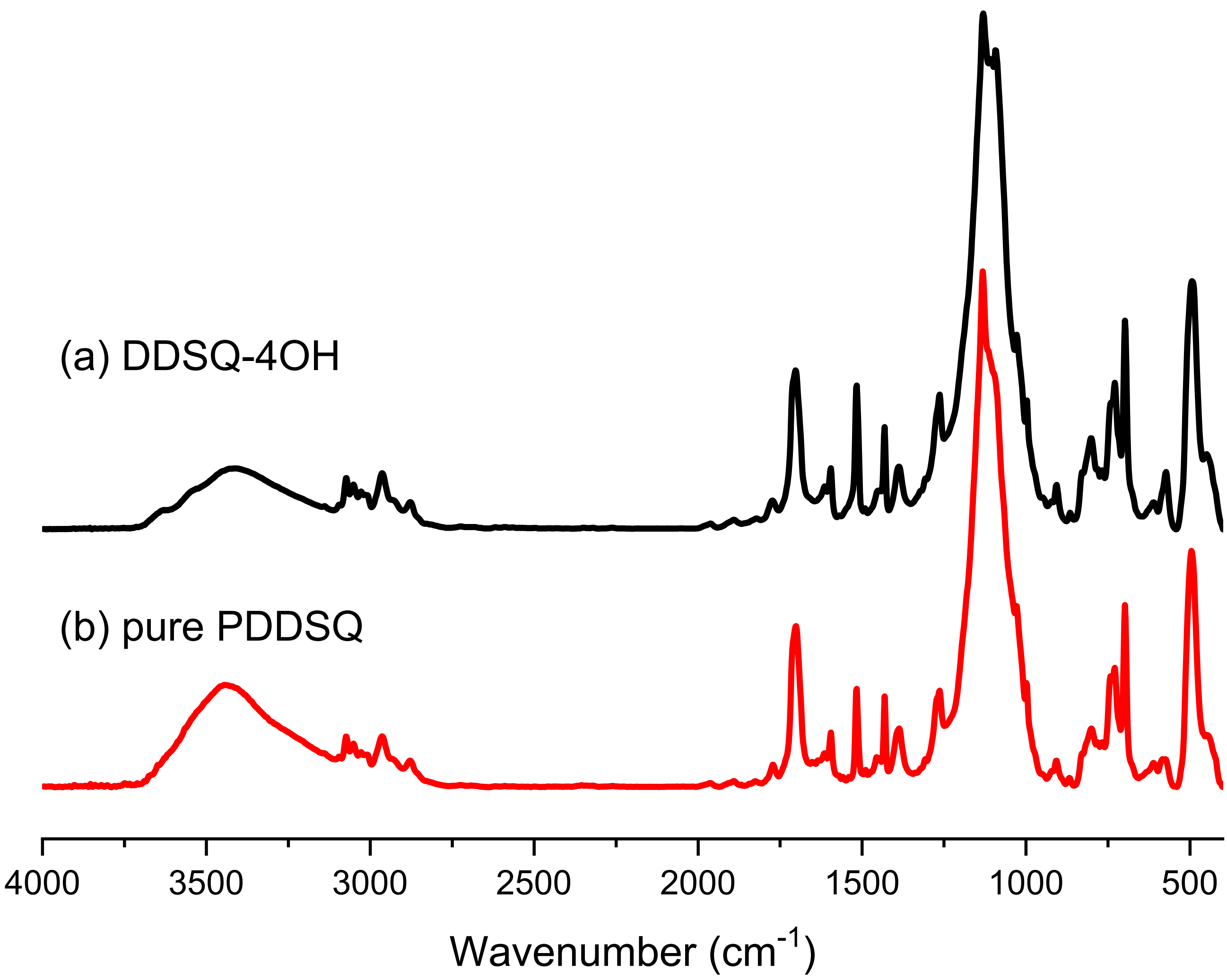

Figure S1. FTIR spectra of (a) DDSQ-4OH and (b) pure PDDSQ.

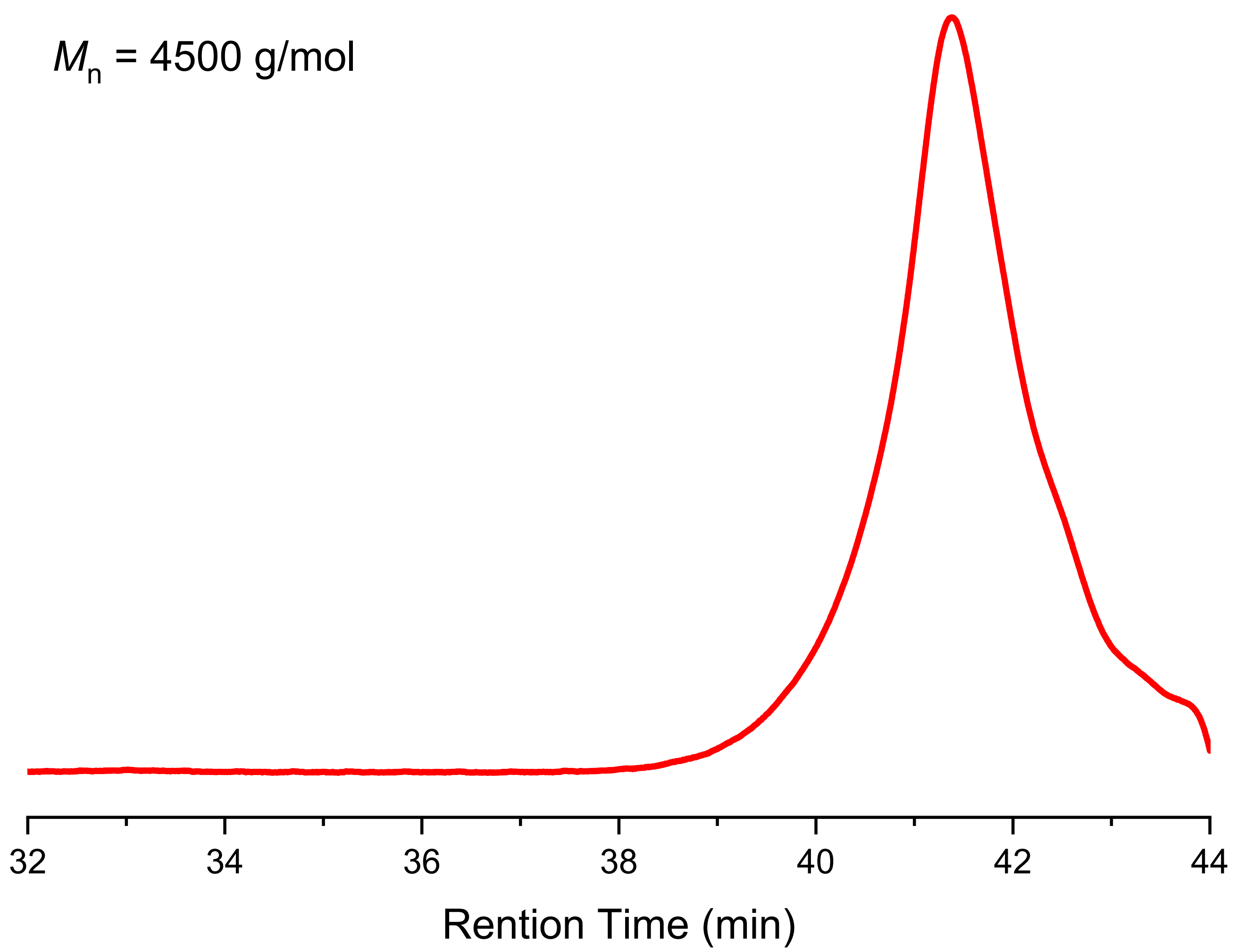

Figure S2: GPC analysis of pure PDDSQ

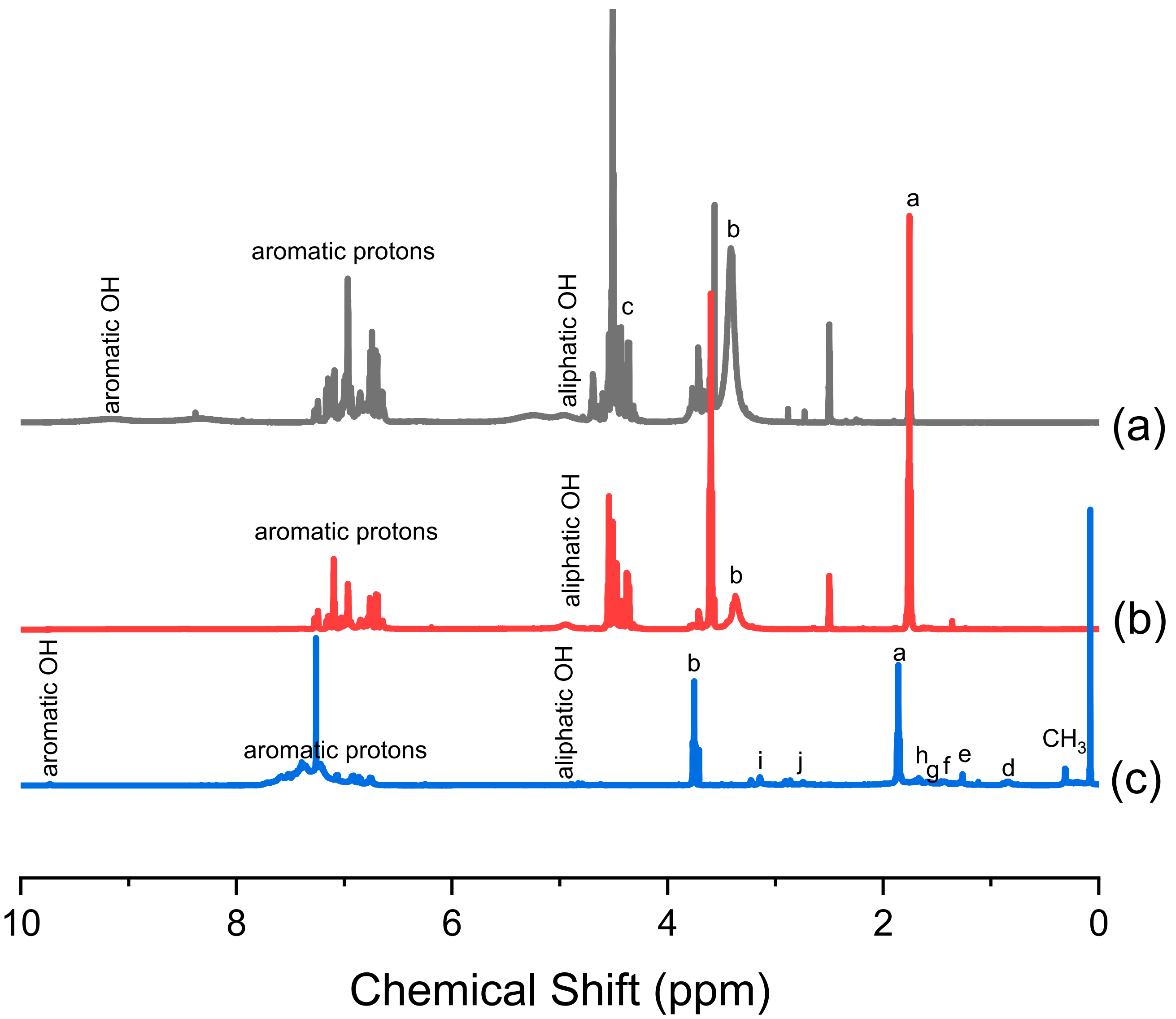

Figure S3: ^{1}H NMR spectra of (a) pure phenolic, (b) PDDSQ-50 and (c) pure PDDSQ.

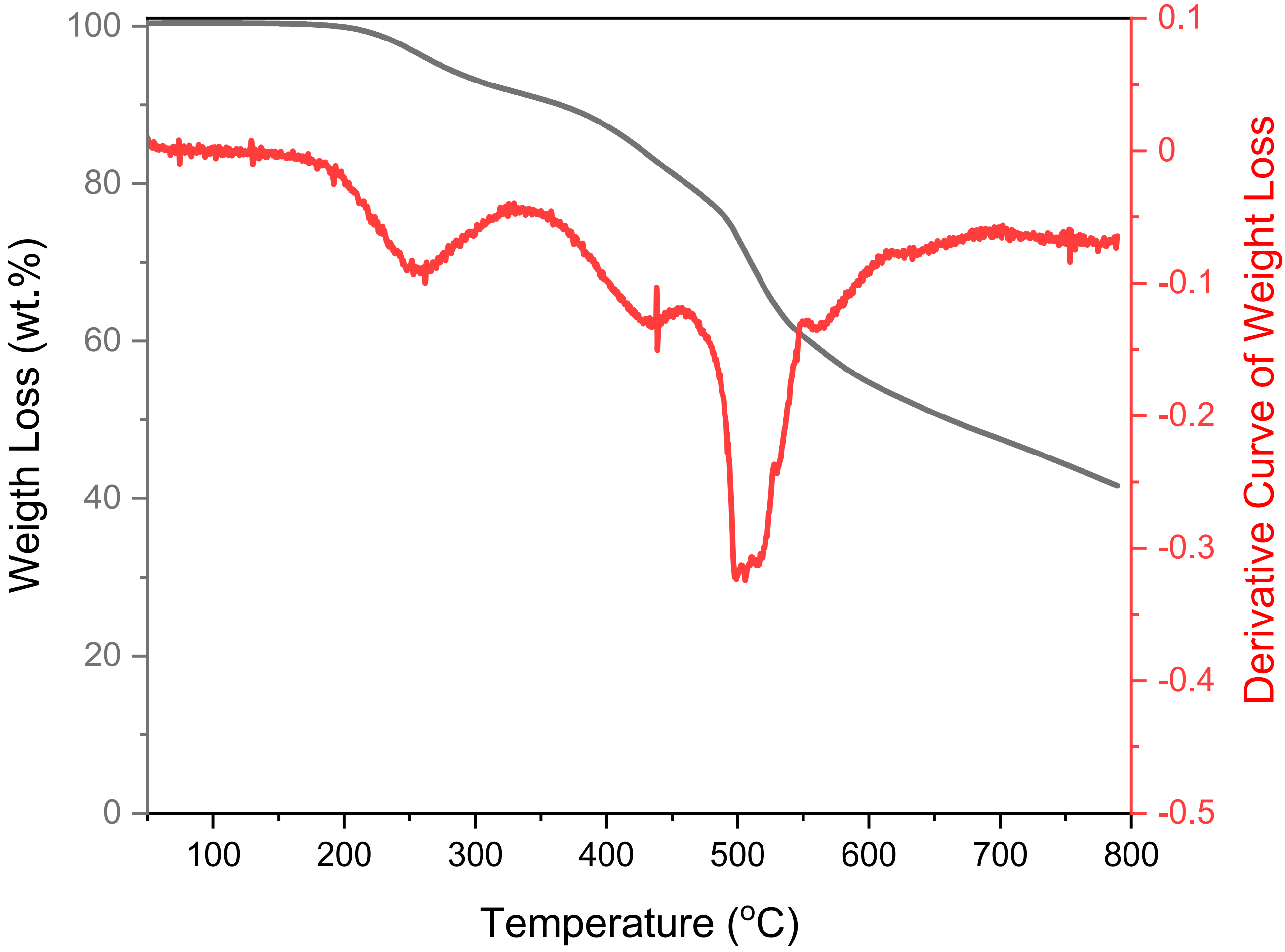

Figure S4: TGA analyses of pure phenolic resin and its corresponding derivative curve.
